# Supplementary material for: Resting behaviour of malaria vectors in highland and lowland sites of western Kenya: Implication on malaria vector control measures
Source: PLoS One. 2020 Feb 25;15(2):e0224718. doi: 10.1371/journal.pone.0224718 (PMC7041793; doi:10.1371/journal.pone.0224718)
Supplement: S1 Table — (DOCX) [file pone.0224718.s001.docx]

**Supplement Table 1.** Summary of female *Anopheles* mosquitoes collected from indoor and outdoor in highland (Bungoma) and lowland (Kisian) settings of western Kenya

| **Study site and *Anopheles* spp.** | | **Indoor** | | | |  | |  | | **Outdoor** | | | | | |  | | **Total** | |  |
| --- | --- | --- | --- | --- | --- | --- | --- | --- | --- | --- | --- | --- | --- | --- | --- | --- | --- | --- | --- | --- |
|  |  | **Aspiration** | | **PSC** | | **Total** | |  | | **Pitshelter** | | **Claypots** | | **Aspiration** | | **Total** | |  | |  |
| **Bungoma**  *An.gambiae s.l*  *An.funestus s.l* | |  | |  | |  | |  | |  | |  | |  | |  | |  | |  |
|  |  | 1136 | | 470 | | 1606 | |  | | 57 | | 41 | | 177 | | 274 | | **1880** | |  |
|  |  | 651 | | 147 | | 798 | |  | | 0 | | 0 | | 28 | | 28 | | **826** | |  |
|  |  |  | |  | |  | |  | |  | |  | |  | |  | |  | |  |
| **Kisian**  *An.gambiae s.l*  *An.funestus s.l* | |  | |  | |  | |  | |  | |  | |  | |  | |  | |  |
|  |  | 288 | | 165 | | 453 | |  | | 167 | | 67 | | 94 | | 328 | | **781** | |  |
|  |  | 28 | | 44 | | 72 | |  | | 0 | | 0 | | 7 | | 7 | | **79** | |  |
|  |  |  | |  | |  | |  | |  | |  | |  | |  | |  | |  |
|  | **Total** | |  | |  | | **2929** | |  | |  | |  | |  | | **637** | | **3566** | |

PSC, pyrethrum spray catch
