# Supplementary material for: Resting behaviour of malaria vectors in highland and lowland sites of western Kenya: Implication on malaria vector control measures
Source: PLoS One. 2020 Feb 25;15(2):e0224718. doi: 10.1371/journal.pone.0224718 (PMC7041793; doi:10.1371/journal.pone.0224718)
Supplement: S2 Table — (DOCX) [file pone.0224718.s002.docx]

**Supplement Table 2.** **Summary results of KDR alleles for indoor and outdoor resting mosquitoes in Bungoma and Kisian sites in western Kenya**

|  |  | **1014S** | | | | |  | **1014F** | | | |
| --- | --- | --- | --- | --- | --- | --- | --- | --- | --- | --- | --- |
|  |  | **NO** | **LL** | **LS** | **SS** | **N/A** |  | **LL** | **LF** | **FF** | **N/A** |
| **Bungoma** |  |  |  |  |  |  |  |  |  |  |  |
| indoor | *An. gambiae s.s.* | 195 | 17 | 4 | 173 | 1 |  | 16 | 4 | 16 | 159 |
|  | *An. arabiensis* | 20 | 18 | 0 | 2 | 0 |  | 12 | 0 | 0 | 2 |
|  |  |  |  |  |  |  |  |  |  |  |  |
| outdoor | *An. gambiae s.s.* | 133 | 19 | 1 | 112 | 1 |  | 20 | 0 | 0 | 113 |
|  | *An. arabiensis* | 32 | 32 | 0 | 0 | 0 |  | 32 | 0 | 0 | 0 |
| **Kisian** |  |  |  |  |  |  |  |  |  |  |  |
| indoor | *An. gambiae s.s.* | 107 | 26 | 3 | 75 | 3 |  | 25 | 2 | 0 | 80 |
|  | *An. arabiensis* | 110 | 90 | 0 | 20 | 0 |  | 95 | 0 | 0 | 15 |
|  |  |  |  |  |  |  |  |  |  |  |  |
| outdoor | *An. gambiae s.s.* | 18 | 7 | 0 | 11 | 0 |  | 5 | 0 | 0 | 11 |
|  | *An. arabiensis* | 78 | 74 | 4 | 12 | 0 |  | 58 | 0 | 0 | 18 |
|  |  |  |  |  |  |  |  |  |  |  |  |
| Bungoma | *An. gambiae s.s.* | 328 | 36 | 5 | 285 | 2 |  | 36 | 4 | 16 | 272 |
|  | *An. arabiensis* | 52 | 50 | 0 | 2 | 0 |  | 44 | 0 | 0 | 2 |
| Kisian | *An. gambiae s.s.* | 125 | 33 | 3 | 86 | 3 |  | 30 | 2 | 0 | 91 |
|  | *An. arabiensis* | 188 | 164 | 4 | 32 | 0 |  | 153 | 0 | 0 | 33 |
